# Detection of *Ehrlichia, Anaplasma*, and *Babesia* spp. in dogs of Cebu, Philippines

**DOI:** 10.14202/vetworld.2018.14-19

**Published:** 2018-01-12

**Authors:** Rochelle Haidee D. Ybañez, Adrian P. Ybañez, Lyra Lee A. Arnado, Laila Monika P. Belarmino, Knowlie Gay F. Malingin, Paul Bien C. Cabilete, Ziggy Ryan O. Amores, Maxfrancis G. Talle, Mingming Liu, Xuenan Xuan

**Affiliations:** 1 College of Science, University of the Philippines Cebu, Gorordo Avenue, Lahug, Cebu City 6000, Philippines; 2 National Research Center for Protozoan Diseases, Obihiro University of Agriculture and Veterinary Medicine, Obihiro City 080-8555, Japan; 3 College of Veterinary Medicine at Barili Campus and College of Arts and Sciences at Main Campus, Cebu Technological University, Cor. M. J. Cuenco Ave. and R. Palma St. Cebu City, 6000 Cebu, Philippines; 4 Gullas College of Medicine, Inc., University of the Visayas, Banilad, Mandaue City 6014, Cebu, Philippines; 5 College of Veterinary Medicine, Southwestern University, Villa Aznar, Urgello St., Cebu City 6000, Philippines; * Equal author

**Keywords:** *Babesia*, Cebu, dogs, *Ehrlichia/Anaplasma*, Philippines

## Abstract

**Background:**

*Ehrlichia*, *Anaplasma*, and *Babesia* spp. are canine pathogens transmitted by the *Rhipicephalus sanguineus* tick which can cause varied clinical signs. These pathogens have been investigated in the Philippines, but coinfection has not been reported yet.

**Aim:**

The aim of this study was to evaluate the presence of *Ehrlichia/Anaplasma* and *Babesia* spp. in Philippine dogs.

**Materials and Methods:**

A total of 100 dogs from seven different veterinary establishments in Cebu, Philippines, were examined for *Ehrlichia/Anaplasma* and *Babesia* spp. infection using peripheral blood smear examination and polymerase chain reaction (PCR). Inclusion criteria included a history or presence of tick infestation, anemia, and/or thrombocytopenia. Clinical signs were recorded. Statistical analyses were performed between PCR positivity and clinical signs and hematological results.

**Results:**

A total of 10 and 18 dogs were found to be positive for *Ehrlichia/Anaplasma* and *Babesia* spp., respectively. One animal was PCR positive for both pathogens, which is the first report of coinfection in the country. The most common clinical signs observed include inappetence (89%), lethargy (80%), thrombocytopenia (85%), and anemia (74%). Analyses revealed that inappetence (p=0.044) and weight loss (p=0.028) were found statistically significant with *Ehrlichia/Anaplasma* infection. Basophil (p=0.001) and eosinophil counts (p=0.000) were also found significantly different between *Ehrlichia/Anaplasma* spp.-positive and -negative dogs. On the other hand, differential monocyte count (p=0.009) was found significantly different between *Babesia* spp.-positive and -negative dogs.

**Conclusion:**

The present study showed low infection rates of canine ehrlichiosis/anaplasmosis and babesiosis and provided additional evidence for the presence of the pathogens in the area.

## Introduction

Canine babesiosis, canine ehrlichiosis (canine monocytic ehrlichiosis [CME]), and canine anaplasmosis are diseases transmitted by the brown dog tick *Rhipicephalus sanguineus* [1-5]. This tick species is also a vector for several other pathogens and coinfection in individual ticks has been shown to occur [6]. Concurrent infections in dogs [7-9] can be fatal [10]. Hence, the detection of coinfection is vital for the institution of appropriate veterinary treatment. Economic losses associated with these diseases can be significant, which include the cost of treatment, abortions, and death.

*Babesia* spp., *Ehrlichia* spp., and *Anaplasma* spp. have global distribution, primarily in tropical and subtropical regions [10-13]. In the Philippines, detection, and diagnosis of *Ehrlichia/Anaplasma* and *Babesia* spp. infections are mostly done through serological testing and peripheral blood smear examination (PBSE) [4,14]. PBSE is the simplest and most accessible diagnostic test for most veterinarians in detecting intraerythrocytic parasites but may be unreliable because pathogens may be absent in blood smears when parasitemia is low [15]. Moreover, accurate identification can be difficult if morphology alone is used as the basis for identification of species. On the other hand, disease diagnosis in chronically infected and carrier dogs remains challenging due to very low and often intermittent parasitemia [16].

While coinfection of *Ehrlichia*/*Anaplasma* and *Babesia* spp. in dogs may be present in other countries, there had has been no previous report yet in the Philippines. Obtaining information on the status of these diseases and their possible coinfection in dogs is important for its surveillance and prompt diagnosis. The present study generally aimed to detect *Ehrlichia/Anaplasma* and *Babesia* spp. infection in dogs using PBSE and polymerase chain reaction (PCR) and to document the presenting clinical signs and hematological values of the suspected dogs in Cebu, Philippines.

## Materials and Methods

### Ethical approval

The procedures performed in this study were guided by the principles of animal welfare, Animal Welfare Act of the Philippines (RA 8485) and Administrative Order No. 45 of the Bureau of the Animal Industry of the Philippines.

### Research design

The study was a prospective descriptive analytical type which involved testing of suspected dogs from selected veterinary clinics for *Ehrlichia*/*Anaplasma* and *Babesia* spp. Profile of dogs, presenting clinical signs, and hematological values were obtained.

### Research subjects and environment

A total of 100 dogs, regardless of sex, age, and breed, which have been suspected for Ehrlichiosis/Anaplasmosis or Babesiosis were selected based on criteria, including the presence or history of tick infestation, thrombocytopenia, and anemia. Dogs were conveniently selected from seven veterinary establishments in Cebu, Philippines, including GPY Veterinare Animale Clinic at Punta Princesa, Cebu City, Southwestern University Veterinary Teaching Hospital (SWU-VTH), Basak, Cebu City, Pet Doctor’s Veterinary Clinic at Talisay City, Animal Kingdom Veterinary Hospital at Lahug, Cebu City, Animal Wellness Veterinary Hospital at Banilad, Cebu City, Pets in the City Veterinary Clinic at Talamban, Cebu City, and Pet Science Veterinary Clinic, at Talamban, Cebu City. The hematological examination was performed at SWU-VTH, while DNA extraction and PCR testing were performed at the Molecular Biology Laboratory of the University of the Philippines Cebu.

### Sample collection and processing

Blood samples were aseptically collected from the peripheral veins using a 3 ml syringe and were separated into aliquots for DNA extraction (in the sterile plain tube) and complete blood count (CBC) analysis (in the sterile ethylenediaminetetraacetic acid tube) and PBSE. Blood samples for DNA extraction were stored at −20°C until further use. DNA extraction, elution, and storage were performed as previously described [1].

### PCR

For *Ehrlichia/Anaplasma* spp. detection, PCR based on 16S rRNA with primer pairs EHR16D and EHR16SR were used following a previously described procedure [17]. For *Babesia* spp. detection, PCR based on 18S rRNA PCR assay with forward (5’- GCATTTAGCGATGGACCATTCAAG -3’) and (5’- CCTGTATTGTTATTTCTTGTCACTACCTC-3’) reverse primers was used [18]. Final volumes were modified to 10 µL and 25 µL for the first and second round PCRs, respectively, using a similar method by [15]. The negative control used was double-distilled water. *Anaplasma phagocytophilum* and *Babesia gibsoni* DNA were used as positive controls. Amplicons were viewed under ultraviolet light illumination using 1.5% agarose gel after electrophoresis.

### Data collection and analysis

Profile, clinical signs, and hematological values of the selected dogs were recorded in a sheet and were encoded in Microsoft Excel using appropriate variable coding. Data were imported into a statistical software. Descriptive statistics were employed. Statistical significance was assessed using Chi-square and Mann–Whitney tests.

## Results and Discussion

Most of the dogs were purebred (56%), with shih-tzu (14%) as the most common breed. The majority were male (56%), with an average age of 2.5 years (standard deviation =2.3 years). Identifying breed, age, and sex predilections can be useful in tick-borne disease (TBD) diagnosis, but several past reports have shown inconsistent results. Some reported that canine ehrlichiosis may have no breed, sex, and age preference [19,20], while others reported that the severity of the disease might be influenced by breed [21,22]. German shepherds are shown to be predisposed to show hemorrhagic signs (including epistaxis) [23], while the beagles and mongrels are believed to show the typical signs of the disease [24]. For canine babesiosis, younger dogs can be more susceptible to *B. vogeli* [25], although it was also suggested that the pathogen could infect dogs at any age groups [26]. Furthermore, crossbred dogs may have higher chances of acquiring the infection than purebred dogs, although there might be no sex predilection [26]. More investigations are needed to determine breed and age predispositions in the Philippine setting.

Although it was expected that a high number of the subjects would be positive because of the inclusion criteria, PCR results revealed that only 10 and 17 dogs were found positive for *Ehrlichia/Anaplasma* and *Babesia* spp., respectively, and with only one animal positive for both pathogens. These results are lower than those reported by Baticados *et al*. [27] and Cruz-Flores *et al*. [14] but similar to that of Corales *et al*. [28]. The difference may be due to the varying place and time of sample collections of the previous studies. The result indicates that unless confirmatory or diagnostic tests are performed, practitioners should not always be biased to canine ehrlichiosis, anaplasmosis, and babesiosis even if some signs may appear to be indicative of these diseases.

The coinfected case reported in this study is the first report in the Philippines. The infected patient was a female mixed bred 3-month-old puppy. The presenting clinical signs were non-specific, including tick infestation, inappetence, fever, and lethargy (Table-1). Thrombocytopenia and anemia were also observed in the infected dog (Table-2). Coinfection with *Ehrlichia/Anaplasma* and *Babesia* spp. in dogs has been observed in other countries. At least one clinical sign that characterizes the different TBDs was observed in these cases [29-31]. The possibility of coinfection of *Ehrlichia/Anaplasma* and *Babesia* spp. or even more than two pathogens is usually higher in thrombocytopenic cases [30,31].

**Table-1:** Clinical signs of dogs PCR positive for *Ehrlichia/Anaplasma* and/or *Babesia spp*. in Cebu, Philippines (n=100).

Clinical signs	*Ehrlichia/Anaplasma* positive (n=9)	Babesia positive (n=16)	Coinfection (n=1)	Negative (n=74)	Total (n=100)
Inappetence	6	12	1	70	89
Tick infestation	9	12	1	67	89
Lethargy	6	13	1	60	80
Pale mucous membrane	1	7	1	30	39
Fever	3	4	1	26	34
Weight loss	0	5	0	26	31
Vomiting	0	2	0	18	20
History of *Ehrlichia*	1	2	0	14	17
Seizure	1	1	0	4	6
Mortality	0	1	0	4	5
Splenomegaly	0	1	0	3	4
Jaundice	1	0	0	0	1
Petechiae and Ecchymosis	0	0	0	1	1
Hemoglobinuria	0	0	0	1	1

PCR=Polymerase chain reaction

**Table-2:** Complete blood count analysis results of dogs tested for *Ehrlichia/Anaplasma* and *Babesia spp*. in Cebu, Philippines (n=100).

Parameter	Reference values	Mean±SD		Co-infection (n=1)	Negative (n=74)
	
		*Ehrlichia/Anaplasma* positive (n=9)	Babesia positive (n=16)		Mean±SD
Packed cell volume (%)	35-57	31.1±9.5	30.0±9.3	36.0	31.3±13.4
RBC (×10^6^/µL)	5.0-7.9	4.9±1.2	4.8±1.6	4.9	4.5±2.1
MCV (fL)	66-77	62.7±4.2	65.3±15.6	74.2	77.2±30.5
Hemoglobin (g/dL)	12-19	10.3±3.0	10.0±3.2	12.1	9.9±4.9
MCH (pg)	21.0-26.2	19.0±2.4	21.7±5.3	24.9	25.5±10.4
MCHC (g/dL)	32-36.3	33.3±0.8	33.2±1.2	33.6	33.5±0.1
Platelet (×10^3^/µL)	211-621	97.0±66.5	108.6±81.5	30.0	130.4±103.0
WBC (×10^3^/µL)	5.0-14.1	14.0±7.5	13.6±8.9	5.9	13.8±11.9
Differential count (%)					
Basophil	0-1	0.0±0.0	0.7±1.4	0.0	0.5±1.6
Eosinophil	0-9	0.0±0.0	0.8±1.3	1.0	1.4±3.1
Neutrophil	58-85	76.1±15.8	63.9±26.4	73.0	71.1±20.3
Monocyte	2-10	4.8±3.9	3.1±2.3	2.0	5.0±4.5
Lymphocyte	8-21	18.9±14.5	30.9±26.9	24.0	21.8±19.5

RBC=Red blood cell, WBC=White blood cell, SD=Standard deviation

None of the blood smears were found positive, which implied low bacteremia and/or parasitemia. The absence of pathogens in blood smears does not rule out the possibility of infection as PBSE has low sensitivity and reliability [4,17]. Although PBSE has limited capability, it is a simple and cheap method to detect TBD pathogens that can be useful, especially when commercial test kits are not available or are deemed expensive. Veterinarians should perform PBSE if condition warrants. However, molecular methods are useful in cases where PBSE is negative and/or clinical signs are not definitive [32].

Almost all of the subjects were exhibiting inappetence (89%) and lethargy (80%) and had tick infestation (89%). Other observed signs included pale mucous membrane (39%), fever (34%), weight loss (31%), and vomiting (20%) (Table-1). The most observed clinical signs remain consistent in those positive for *Ehrlichia/Anaplasma* and/or *Babesia* spp. These clinical signs are non-specific and can also be observed in other TBDs [33]. For *E. canis* cases, expression of clinical signs may be partly caused by the infection of the pathogen to circulating monocytes that will affect different body systems or organs producing varied clinical signs or their combinations [3,34]. On the other hand, the chronic cough and hematuria observed in *Babesia* spp.-infected dogs [33] were not observed. The varying signs may be caused by indefinite clinicopathological patterns [25] or by the disease stages of the infected animals [35].

Including the coinfected, 13 of the 17 *Babesia* spp. positive had tick infestation and all (10 of 10) of the *Ehrlichia/Anaplasma* spp. had tick infestation. For those positive but with no observed ticks, the dogs may have been exposed to the ticks and pathogens earlier. This finding is similar to a previous study where the absence or presence of ticks in the observed patient did not rule out the possibility of infection [13,36].

The reported clinical signs of the subjects will give suspicion for an *Ehrlichia/Anaplasma* and/or *Babesia* spp. infection because these signs are common for TBDs. These signs have been reported in canine ehrlichiosis cases [18,37], with the addition of other signs, including apathy, lymphadenopathy, splenomegaly, and uveitis. Epistaxis, which is the most dramatic sign of CME experimental infection in German shepherd dogs [34], was not observed in this study.

CBC results revealed that 85%, 69%, and 55% were found anemic, thrombocytopenic, and both, respectively. Similar to the previous studies [13], anemia and thrombocytopenia were the most common hematologic observations in the *Ehrlichia/Anaplasma* and *Babesia* spp.-positive dogs (Table-2). The mean PCV and red blood cells (RBC) counts were lower than the reference values. Although inconsistent, these findings were similar in experimentally *E. canis*-infected dogs where low PCV and RBC were observed [38]. Similarly, platelet counts were lower than the reference values which may be due to the presence of antiplatelet serum during CME infection [39]. Platelet count is a good screening test for *E. canis* infection [40] and is used to assess recovery from the disease [41]. On the other hand, it was expected that *Babesia* spp.-infected animals would exhibit anemia and paleness because these pathogens parasitize RBCs [25,42,43]. These observed hematological signs and the non-specific clinical signs observed in the subjects found negative for the tested pathogens which can be characteristic of other TBD pathogens such as *Rickettsia*, *Mycoplasma, Hepatozoon*, and *Bartonella* spp. [44], the low detection of *Ehrlichia/Anaplasma* and *Babesia* spp., and the presence of the common tick vector in the area suggest the possible presence of other TBDs in the studied patients. Further investigations are needed to determine their epidemiologic status in the country.

Statistical analyses revealed that inappetence (p=0.044) and weight loss (p= 0.028) were found statistically significant with *Ehrlichia/Anaplasma* infection. Basophil (p=0.001) and eosinophil counts (p=0.000) were also found significantly different between *Ehrlichia/Anaplasma* spp.-positive and -negative dogs. Basophil counts were within the normal range, but those from *Anaplasma/Ehrlichia* spp.-negative dogs were mostly higher than those positive, which conflicts the previous findings [13,45] where *Anaplasma platys*-infected dogs had higher basophil counts. Similarly, eosinophil counts were also higher in negative dogs. Further studies are needed to clarify these observations. On the other hand, differential monocyte count (p=0.009) was found significantly different between *Babesia* spp.-positive and -negative dogs. Similar to a previous study [46], average monocyte counts in this study were within the normal range, but these were observed to be lower in *Babesia* spp.-positive dogs (3.0%) than those negative (5.0%). Further investigation is also needed to clarify this observation.

The *R. sanguineus* tick can host several pathogens at a time, which can result in coinfection [47-49]. Coinfection with several pathogens is common [50], producing severe and fatal signs in dogs [51,52]. Due to possibilities of coinfection [53], especially with other TBD pathogens that were not tested in this study, it may be difficult to associate a specific clinical sign or hematological abnormality to a particular canine vector-borne disease.

Canine TBDs continue to be global threats. With the common clinical signs seen in several TBDs either as sole or coinfection, its diagnosis and treatment are challenging. Surveillance of possible TBD pathogens in an area is important, especially when the vector is ubiquitous. This will assist veterinary practitioners in making differential diagnoses. As the present study only tested for *Ehrlichia/Anaplasma* and *Babesia* spp., it cannot be ruled out that the observed clinical signs might be influenced by other pathogens. It will be interesting to test for other TBD pathogens, including *Rickettsia*, *Bartonella*, and *Hepatozoon* spp., in the future and assess their clinical signs most, especially, in coinfections.

## Conclusion

*Ehrlichia/Anaplasma* and *Babesia* spp. were molecularly detected in dogs in Cebu, Philippines. One dog was found coinfected with both pathogens, which is the first report in the country. The most common clinical observations include tick infestation, lethargy, inappetence, anemia, and thrombocytopenia. The results of this study posit the possible presence of other TBD pathogens in the country.

## Authors’ Contributions

RHDY and APY conceptualized the study and analyzed and wrote the manuscript. LLAA, LMPB, KGFM, PBCC, ZROA, and MGT took charge of the sample collection and data analyses. ML and XX gave valuable insights and support in the conduct of the study. All authors finally read and approved the final manuscript.
